# Hydrogel-Encapsulated Beads Enable Proximity-Driven Encoded Library Synthesis and Screening

**DOI:** 10.1021/acscentsci.3c00316

**Published:** 2023-07-13

**Authors:** Valerie Cavett, Alix I Chan, Christian N. Cunningham, Brian M. Paegel

**Affiliations:** † Department of Pharmaceutical Sciences, University of California, Irvine, California 92697, United States; ‡ Department of Peptide Therapeutics, Genentech, South San Francisco, California 94080, United States; § Department of Pharmaceutical Sciences, University of California, Irvine, California 92697, United States; ∥ Departments of Chemistry & Biomedical Engineering, University of California, Irvine, California 92697, United States

## Abstract

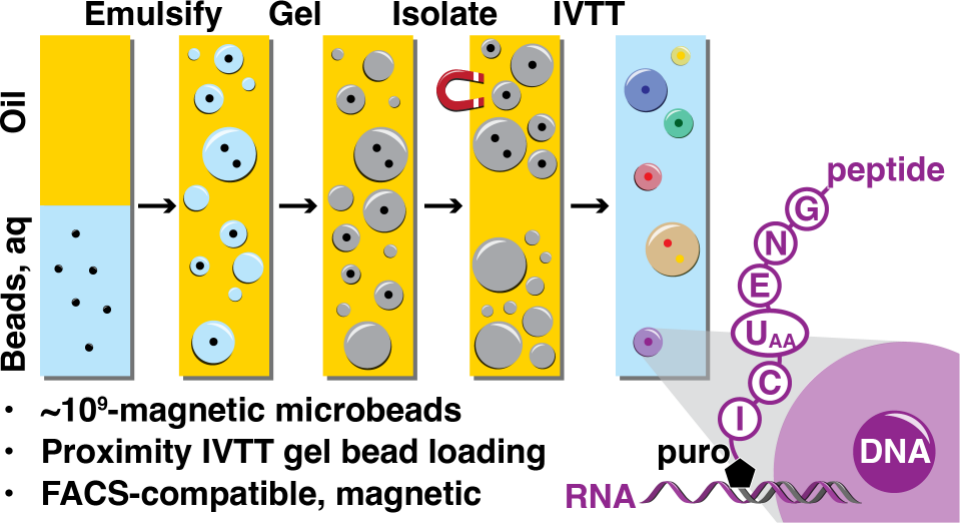

Encoded combinatorial library technologies have dramatically expanded the chemical space for screening but are usually only analyzed by affinity selection binding. It would be highly advantageous to reformat selection outputs to ”one-bead-one-compound” solid-phase libraries, unlocking activity-based and cellular screening capabilities. Here, we describe hydrogel-encapsulated magnetic beads that enable such a transformation. Bulk emulsion polymerization of polyacrylamide hydrogel shells around magnetic microbeads yielded uniform particles (7 ± 2 μm diameter) that are compatible with diverse in-gel functionalization (amine, alkyne, oligonucleotides) and transformations associated with DNA-encoded library synthesis (acylation, enzymatic DNA ligation). In a case study of reformatting mRNA display libraries, transcription from DNA-templated magnetic beads encapsulated in gel particles colocalized both RNA synthesis via hybridization with copolymerized complementary DNA and translation via puromycin labeling. Two control epitope templates (V5, HA) were successfully enriched (50- and 99-fold, respectively) from an NNK_5_ library bead screen via FACS. Proximity-driven library synthesis in concert with magnetic sample manipulation provides a plausible means for reformatting encoded combinatorial libraries at scale.

## Abstract 2

Bead-templated gel particles via emulsion polymerization can scalably transform display-type encoded libraries into one-bead-one-compound libraries suitable for functional screening.

## Introduction

Combinatorial synthesis offers unprecedented access to novel, expansive chemical spaces. Encoded combinatorial library technologies, such as DNA-encoded libraries (DEL)^1,2^ and peptide mRNA display^3,4^ in particular, are playing increasingly important roles in early drug discovery for their ability to generate very large compound collections (10^6^–10^14^ members) with sometimes highly unconventional physicochemical properties. These ”beyond rule of 5” (bRo5)^5,6^ libraries are proving highly productive for discovering ligands of transcription factors, protein–protein interactions, and other difficult targets.^7−10^ However, encoded library analysis is almost exclusively limited to binding affinity selections against the purified target and achieving cellular activity starting from affinity selection hits frequently requires intensive additional rounds of optimization.

Implementation of solid-phase tactics for library synthesis has proven effective for expanding into other functional screening modalities. “One-bead-one-compound” (OBOC) split-and-pool synthesis procedures^11,12^ yield combinatorial libraries that are spatially isolated, clonal populations of a single library member that can be individually assayed. The OBOC principle was applied to DEL technology,^13^ and the resulting libraries could be functionally screened using biophysical, biochemical, or cellular assays.^14−16^ For biophysical and biochemical screening, microfluidic compartmentalization was critical for scalably isolating single library beads for activity assay; however, there are high entry barriers to implementing these approaches.

Significant recent technology development efforts have aimed to reduce these barriers. For example, particle-templated emulsions eliminate the need for local droplet microfluidics capabilities.^17,18^ Water-in-oil-in-water (w/o/w) emulsions similarly dodge the need for microfluidic sorting by enabling emulsion screening via commercially available FACS instruments, which has found use in enzyme engineering, high-throughput enzymology, and other important applications.^19−21^ Droplets themselves are difficult to manipulate and can be replaced with hydrogels,^22^ which exhibit solution- and solid-like properties, a key property that spurred their use in single-cell sequencing workflows,^23^ bacterial microcultures,^24^ directed evolution,^22^ and other applications^25^ that require the delivery and confinement of reagents in small volumes. These properties collectively suggested that hydrogel particles could be highly useful for expanding solid-phase OBOC encoded library synthesis capabilities.

Here, we describe a versatile hydrogel-based particle that supports both chemical and biochemical synthesis. Particle preparation via bulk magnetic microbead-templated emulsion polymerization is straightforward, scales well, and yields unusually uniform products. The hydrogel layer can display diverse chemical functionality commonly used in both conventional split-and-pool combinatorial chemical synthesis or templated enzymatic biosynthesis.^26−28^ Templated library preparation via in vitro transcription/translation (IVTT) was particularly advantageous because RNA transcripts and translated peptides localized to their host particle, generating monoclonal beads for screening without further emulsification or other partitioning. We applied this approach to library screening using fluorescence-activated cell sorting (FACS) instrumentation, successfully isolating two control epitopes translated using the conventional 20 biogenic amino acids and library members translated from a recoded genetic table containing the unnatural amino acid azidolysine. This new particle format introduces important handling advantages that will enable encoded library synthesis automation while also unlocking functional screening capabilities by way of a polyvalent encoded library member display.

## Results and Discussion

**Figure 1:**
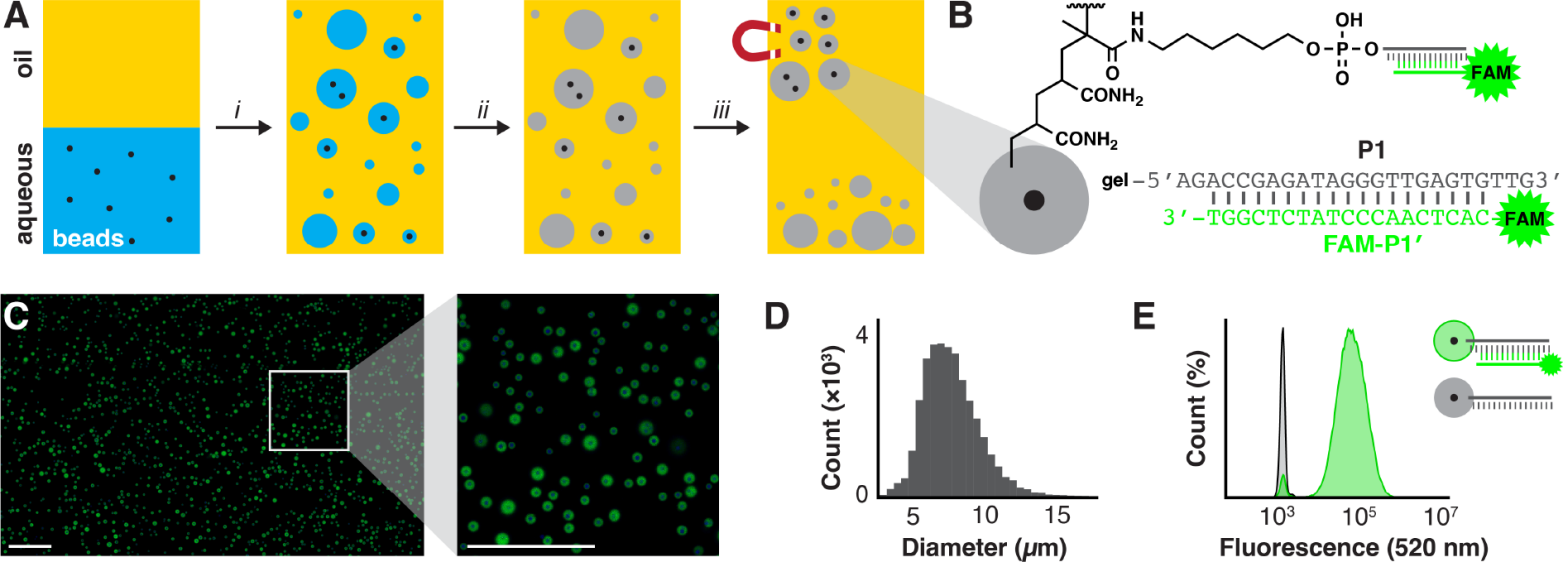
(A) Magnetic beads (2.8 μm, 5 × 10^7^) were suspended in a monomer premix, a layer of catalyst-containing oil was added, and the suspension was emulsified (i), droplets were cured to form hydrogel droplets (ii), and bead-containing gel particles were isolated magnetically (iii). (B) 5′-Methacrylamide-functionalized oligonucleotide (20 μM, P1) was copolymerized in the hydrogel particles and probed with FAM-P1′. (C) Particle size uniformity was analyzed by confocal fluorescence microscopy. Scale bar: 100 μm (D) Diameters were measured for 32000 particles (median 7.3 μm). (E) Flow cytometry (λ_ex_/λ_em_ = 488/520 nm) was used to analyze probed (green) and naive (gray) gel particles.

Hydrogel particles were prepared via emulsification of an acrylamide:bis-acrylamide monomer solution and magnetic microbeads. Magnetic beads were suspended in a monomer solution and combined with catalyst (TEMED)-containing oil, the suspension was emulsified using a bead mill homogenizer and polymerized on ice, and magnetic-bead-containing particles were isolated on a magnet (Figure 1A). Copolymerization with a 5′-methacrylamide-modified DNA oligonucleotide P1 (acrydite)^29^ yielded P1-functionalized gel particles (Figure 1B), which were hybridized with a 5′-fluorescein (FAM)-labeled DNA oligonucleotide complementary to P1 (FAM-P1′) and analyzed by confocal fluorescence imaging (Figure 1C). Over nine preparations (32000 sampled particles), we observed a median diameter of 7 ± 2 μm (Figure 1D). Flow cytometry analysis of the FAM-P1′-probed gel particles yielded a distinct population of signals that were 100-fold increased in fluorescence intensity compared to unprobed gel particles and indicated that >95% of magnetic beads were encapsulated in hydrogel (Figure 1E). Hydrogel particle preparation and analysis were compatible with several different magnetic bead sizes (1, 2.8, and 10 μm diameters). Increasing the magnetic bead size formed larger and more uniform gel particle distributions as shown by imaging microscopy (Figure S1), and all bead sizes yielded populations readily gated by forward and side scatter in flow cytometry for comparison of hydrogel fluorescence upon probing (Figure S2).

Microbead-templated emulsion polymerization yielded surprisingly uniform particles that did not require microfluidics for either preparation or analysis, and the particles exhibited superb handling and biocompatibility. The particle preparation scale ranged from 10^5^ to 10^8^ particles in microtube-compatible reactions and using standard laboratory equipment. Magnetic isolation post curing ensured that only microbead-containing emulsion droplets were retained, the most likely factor enhancing particle uniformity. The gel layer combined with magnetic handling also proved to be very useful for rapid and potentially automatable reagent exchange and bead washing. We chose polyacrylamide specifically because it is easily synthesized and inhibits aggregation and adherence to tube walls. Polyacrylamide is also inert and thus compatible with a range of different chemical and biochemical reactivities.

### Gel Particle Functionalization through Copolymerization of Diverse Methacrylated Moieties

**Figure 2:**
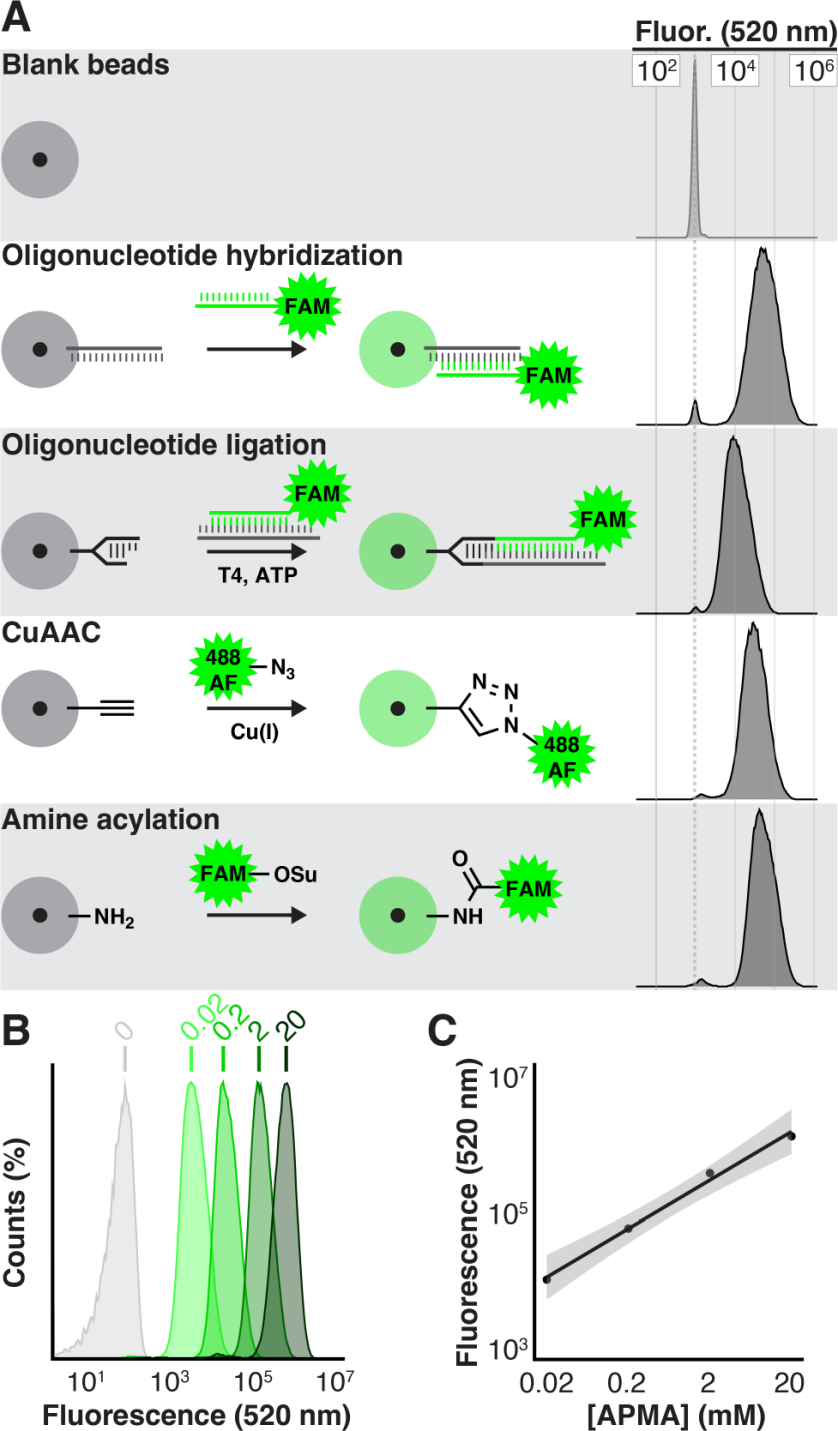
Examples of gel particle functionalization by copolymerization. (A) Gel particles were copolymerized with 5′-methacrylamido oligonucleotide, methacrylamide-modified headpiece DNA (HDNA), propargyl methacrylate, or APMA (20 μM additive). Gel functionalization was detected via fluorescein-labeled complementary oligonucleotide hybridization, enzymatic ligation of fluorescein-labeled dsDNA modules used for DEL synthesis, CuAAC with Alexa Fluor 488 azide (488AF-N_3_), or amine acylation with fluorescein succinimidyl ester (FAM-OSu). Gel labeling was detected via flow cytometry and compared to blank gel particles (right). (B) Gel particles were prepared with varying concentrations of APMA (0.02–20 mM), acylated with FAM-OSu, analyzed by flow cytometry, and (C) quantitated (gray boundary indicates standard error).

We next sought to demonstrate the diversity of functionalization that polyacrylamide hydrogel particles could support via copolymerization. We encapsulated 2.8 μm magnetic beads in hydrogels displaying various chemical functionalities (Figure 2A), such as oligonucleotides using either commercially available 5′-acrydite modification or amino oligonucleotides modified with methacrylic acid (Figure S3), alkynes using propargyl methacrylate (PMA), or primary amines using 3-(aminopropyl)methacrylamide (APMA). Particle functionalization was detected by flow cytometry with the addition of the appropriate fluorescent-dye-labeled substrates, including complementary oligonucleotide, double-stranded oligonucleotide ligation module, azide, or succinimidyl ester, respectively. Depending on reactivity, the relative fluorescence increased between 5- and 25-fold compared to naive gel particles. Hybridization and amine acylation resulted in the largest shift; all functionalization reactions yielded baseline separation of product and starting material by flow cytometry. Hydrogels with escalating amine loading capacity were prepared by copolymerizing increasing concentrations of APMA (0.02–20 mM); acylation with FAM-OSu resulted in log-linear increases in gel particle fluorescence over the 4 order of magnitude serial dilution (Figure 2B,C).

Hydrogels were diversely functionalized by including substoichiometric methacrylamide-modified additives in the acrylamide:bis-acrylamide monomer solution for copolymerization. Incorporation of the synthetic hairpin headpiece DNA (HDNA) and subsequent proof-of-concept enzymatic oligonucleotide ligation reaction demonstrate the viability of standard DEL synthesis workflows using these particles.^1^ Of note, the commercially available NH_2_-HDNA is readily transformed to the methacrylamide analogue for copolymerization. An amine functionality is routinely employed in solid-phase synthesis (and also DEL synthesis). Here, we show that particle loading capacity is quantitative over 4 orders of magnitude. At the highest site density (20 mM), each median diameter (7 μm dia) particle harbors ∼4 fmol amine sites. If this gel particle were used as a vehicle to dose a microfluidic droplet (40 pL),^30^ the resulting maximum concentration would be 100 μM. For in-gel assays,^31^ the same 100 μM concentration is on the lower end of the [APMA] particles tested. Increasing [APMA] > 20 mM inhibited emulsion formation and [oligonucleotide] > 20 μM would be both impractical and unnecessary for routine biochemical transformations.

### In-Gel Transcription of Microbead-Bound DNA Templates Is Proximity Driven, Yielding Polyvalent mRNA-Functionalized Peripheral Gel

**Figure 3:**
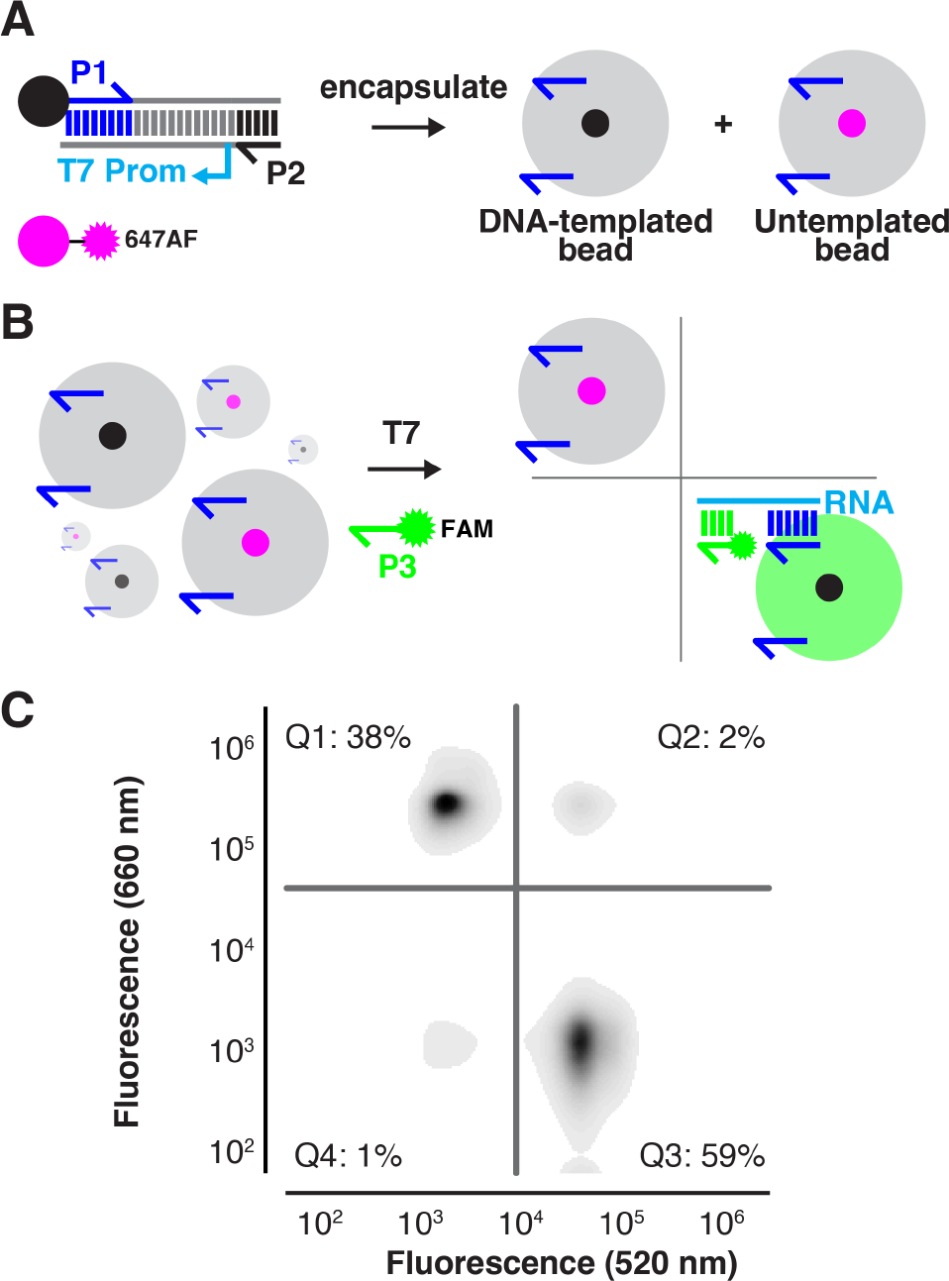
In-gel transcription. (A) DNA-templated beads were prepared by PCR using primer P1 (blue arrow) functionalized beads and primer P2 (black arrow). DNA templates contained a T7 RNA polymerase promoter element (T7 prom, cyan arrow). Untemplated negative control beads were labeled with Alexa Fluor 647 (647AF, magenta). Each bead set was encapsulated in polyacrylamide hydrogel copolymerized with P1. (B) Bulk in vitro transcription of the encapsulated beads in the presence of FAM-labeled oligonucleotide probe of the RNA transcript (RNA probe, green) was expected to yield untemplated beads and templated beads bearing RNA transcript (cyan) hybridized to gel-bound P1 primer and RNA probe. (C) Two-dimensional flow cytometry analysis (contour plot) showed that the majority of particles exhibited either exclusively red fluorescence (untemplated negative controls in Q1) or green fluorescence (templated and RNA-loaded beads in Q3).

While enzymatic ligation plays an important role in the preparation of DNA-encoded chemical libraries, DNA transcription is central in the preparation of genetically encoded RNA and peptide libraries. We hypothesized that RNA transcribed from bead-bound DNA templates could be captured in the peripheral hydrogel if the RNA contained a sequence complementary to the gel-bound oligonucleotides.^32^ DNA-templated 2.8 μm magnetic beads were mixed with untemplated, dye-labeled (Alexa Fluor 647, 647AF) beads as negative controls (Figure 3A). RNA transcripts were detected by including a FAM-labeled DNA oligonucleotide probe of the RNA (Figure 3B). After in vitro transcription of the bead mixture, flow cytometry analysis indicated the presence of 3 populations: 38% high 647AF fluorescence (Q1), 2% fluorescence in both channels (Q2), and 59% high FAM fluorescence (Q3).

The flow cytometry results confirmed our hypothesis that RNA could be captured by hybridization in the gel periphery of the magnetic bead. More significantly, the results showed that migration of transcripts onto untemplated beads occurred infrequently but as a distinct population. This mock experiment represented a worst-case scenario in that it involved only one template sequence and comprised half of the total sample. During library preparation, such cross-contamination would not be an issue since templates would be much more heterogeneous and thus unlikely to populate other beads with any one sequence. More importantly, postscreening PCR would only amplify the bead-bound DNA, thus excluding such cross-talk sequences from both subsequent rounds of directed evolution and final hit analysis.

### In-Gel Translation Is Proximity Driven, Yielding Polyvalent Particles with Colocalized mRNA and Translated Peptide

**Figure 4:**
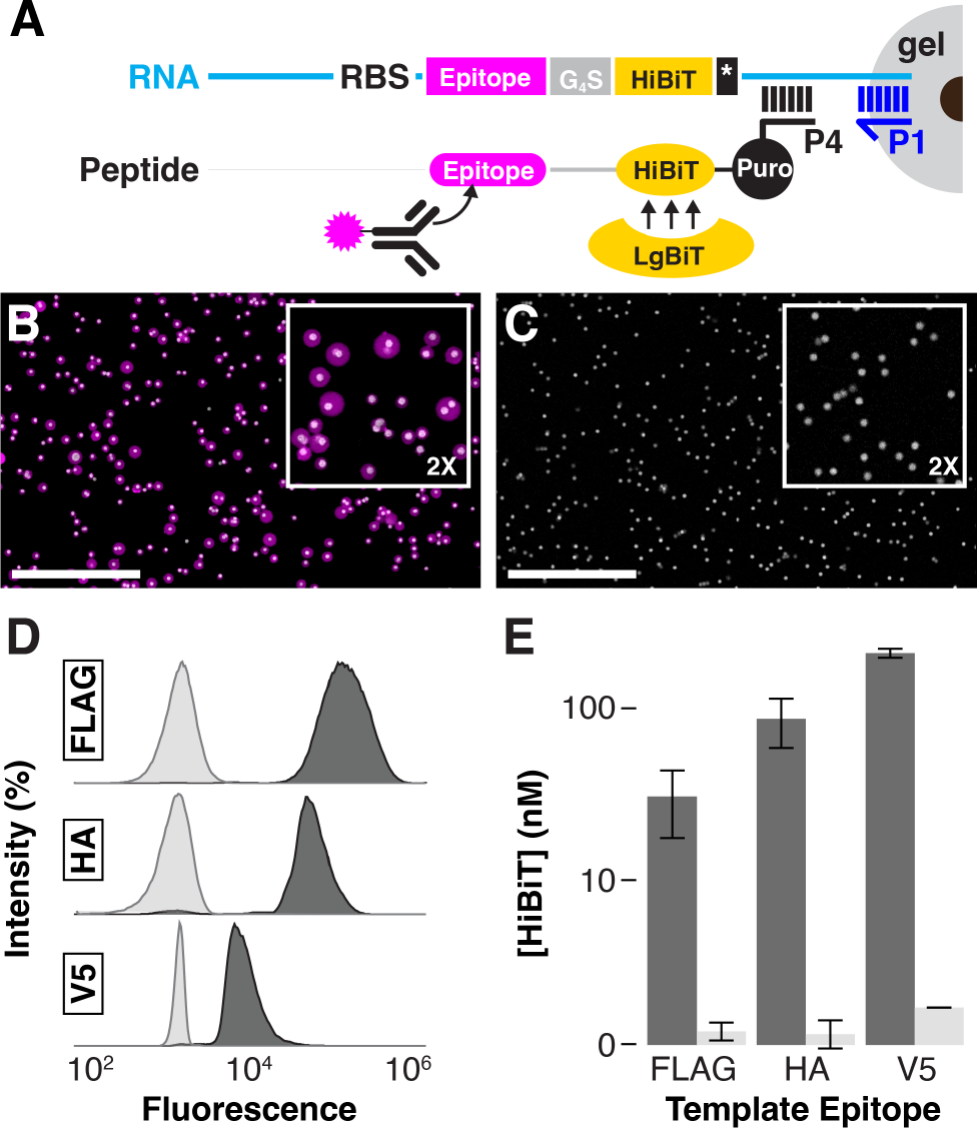
Proximity-driven in-gel translation. (A) DNA-templated beads were encapsulated in polyacrylamide hydrogel copolymerized with P1 (blue). Transcribed mRNAs (cyan) hybridize to gel-linked P1 and a 3′ puromycin-modified DNA oligonucleotide (P4, black). The mRNA encodes a ribosome binding site (RBS), epitope tag (magenta), glycine-serine linker (G4S), HiBiT tag (yellow), and stop codon (*). Ribosomal protein synthesis terminates with incorporation of the 3′ puromycin into the nascent chain, tethering the translated peptide to P4. Epitopes were detected via immunofluorescence, and HiBiT was quantitated via luciferase complementation with LgBiT. (B) In-gel translation of FLAG-templated particles was probed using 647AF-labeled anti-FLAG antibody, visualized, and compared with (C) untranslated particles in confocal imaging of antibody fluorescence (λ_ex_/λ_em_ = 650/720 nm) and magnetic bead autofluorescence λ_ex_/λ_em_ = 490/560 nm). Scale bar: 50 μm. (D) IVTT reactions were programmed with FLAG-, HA-, or V5-templated particles, and translated epitopes were detected with 647AF-anti-FLAG, APC-anti-HA, or CF488A-anti-V5 immunofluorescence by flow cytometry (FLAG and HA, λ_ex_/λ_em_ = 640/660 nm; V5, λ_ex_/λ_em_ = 488/530 nm; translated particles in charcoal, naive particles in light gray). (E) In-gel capture of translated FLAG, HA, and V5 epitopes with and without 10 μM P4 (charcoal, light gray) was quantitated via HiBiT complementation. Error bars reflect the standard deviation of the mean.

We next explored whether in-gel translation was also possible and whether peptide products could be localized analogously to RNA transcripts. Beads were templated with DNA encoding various affinity tag epitopes (FLAG, HA, or V5) fused to a HiBiT luciferase complementation sequence^33^ and encapsulated in P1-functionalized gels. The gel particles were subjected to mRNA display-type^34^ IVTT reactions incorporating a puromycin-modified peptide capture oligonucleotide P4 complementary to the RNA directly 3′ of the stop codon (Figure 4A). Following IVTT reactions, particles were analyzed by imaging microscopy and flow cytometry to visualize specific epitope translation. Gel particles exhibited homogeneous antibody-derived fluorescence throughout the gel periphery (Figure 4B); only magnetic bead autofluorescence was detected in the same particles before translation (Figure 4C). Translated gel particle fluorescence via flow cytometry was baseline-separated compared to untranslated particles for all three example epitopes (Figure 4D). In-gel peptide capture yield was quantitated via an HiBiT luminescence-based in vitro translation efficiency assay.^35^ Particles translated in the presence of puromycin capture oligonucleotide P4 retained ∼100 nM HiBiT peptide, while <1 nM was retained in translations lacking P4 (Figure 4E).

Orthogonal detection of multiple epitope tags demonstrated successful translation of gel-immobilized RNAs and subsequent immobilization of the translation product in the gel particle. Immunofluorescence measurements confirmed the presence of each epitope by specific epitope labeling, also demonstrating a proof-of-concept for conducting in-gel protein binding assays. HiBiT detection quantitatively confirmed the presence of captured peptide with high sensitivity. Because the HiBiT tag is C-terminal, quantitation reflects the minimum concentration of translated peptide retained in the hydrogel particle; N-terminal truncates or other translation byproducts would not be detected. In-gel peptide capture yields were sufficient for robust detection in both immunofluorescence and HiBiT assays, but they fell well short of the RNA capture oligonucleotide P1 concentration (50 μM), which is the limiting reagent for translation product capture; we would expect a 50 μM maximum concentration if all capture sites were occupied by translation product.

### Gel Particle Library Screening via Conventional FACS Analysis

**Figure 5:**
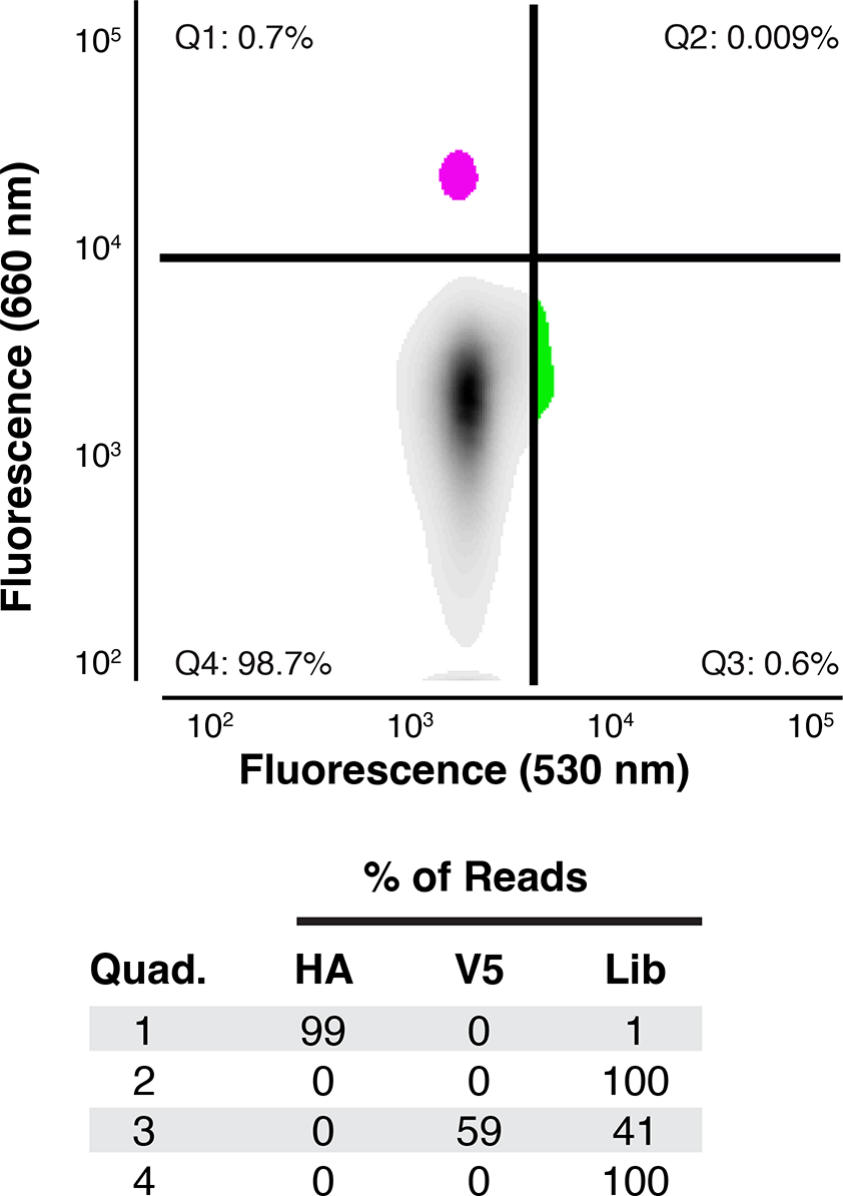
Library screening by FACS. HA and V5 templated particles were spiked (1% each) into a background of library particles (NNK_5_). The particles were then translated in a bulk IVTT reaction and incubated with APC-labeled anti-HA and CF488A-labeled anti-V5 antibodies before FACS. After sorting, each population was PCR-amplified and sequenced. The fraction of reads (%) is tabulated according to quadrant (Quad.) and pattern matching to epitope tag sequences (HA or V5) or NNK_5_ library (Lib).

As a final demonstration of the novel gel particle format’s utility, we explored its compatibility with FACS analysis as a high-throughput screening strategy. A peptide library template with 5 NNK (where N = A/C/G/U, K = G/U) degenerate codons (NNK_5_) fused to the HiBiT tag was used to template magnetic beads via limiting dilution emulsion PCR (emPCR). Single-bead qPCR analysis^36^ of the templated library beads indicated 42000 DNA templates per bead with ∼30% of beads templated (Figure S6). DNA-templated beads were next encapsulated in hydrogels copolymerized with oligonucleotide P1. Library beads (5 × 10^6^) were combined with control epitope beads (1% HA or V5), translated in the presence of puromycin oligonucleotide P4, probed with dye-labeled antibodies (anti-HA-647AF; anti-V5-CF488A), and sorted by FACS into four populations (Figure 5). Sorted hit particles were sequenced to determine enrichment rates for the various control tags. Reads were pattern-matched to epitope tag sequences or the NNK_5_ library degenerate sequence. The HA positive hit pool was 99% HA-encoding sequences, and the V5 hit pool was 50% V5-encoding sequence, 100-fold and 50-fold enrichments from the library starting material, respectively.

We also sought to establish the compatibility of library-scale synthesis and sorting with engineered genetic codes. Beads were functionalized with a DNA template that encodes a single GAC codon (native Gln codon) fused with the HiBiT tag and encapsulated in P1-functionalized gels. An engineered IVTT mix, which lacked glutamine and GlnRS and contained noncanonical amino acid azidolysine (AzK) charged onto CUG-tRNA^Asn^_GGC_ using the dFx flexizyme, was used to display the azide functionality via IVTT.^37−39^ HiBit quantitation yielded levels of captured peptide (105 ± 7 nM) similar to those of the all-natural peptide epitopes previously tested (Figure 4E).^35^ Next, a peptide library template with 5 NNT degenerate codons (where N = A/C/G/T, NNT_5_) was used to template magnetic beads via emPCR, resulting in particles with an average of 2 × 10^4^ template molecules per bead. The NNT degeneracy excludes GAC codons in the NNU_5_ transcripts. After gel encapsulation, we combined NNT_5_ library beads (3 × 10^6^) with AzK-templating beads (∼0.1% of beads) and performed in-gel transcription/translation using the ΔGln/AzK engineered IVTT mix in the presence of P4. After translation, we performed in-gel CuAAC on the entire library mixture with 647AF-alkyne to label AzK-presenting particles and then sorted the 647AF-high population (∼2%) via FACS. After sequencing and decoding, we observed an ∼6-fold enrichment of GAC-templating sequences in the sorted population compared to the starting library pool (Table S2).

These experiments established the feasibility of FACS-based high-throughput screening of gel particle libraries. Using standard PCR protocols for library generation, magnetic beads templated with several control epitope-coding sequences and doped into a conventional library as background were successfully sorted as hits and exhibited substantial enrichment compared to library input. Importantly, coupled transcription and translation of the mixed library and epitope particles without additional compartmentalization yielded sufficiently pure in-gel epitope display for immunofluorescence-based screening. The translation products, like the RNA transcripts, are concentrated in the gel matrix of the DNA-templated magnetic bead, proving that all enzymatic steps are proximity driven.

Magnetic core hydrogel particles support diverse functionalization via copolymerization and subsequent chemical and biochemical procedures analogous to solid-phase synthesis. Particle preparation involves commercially available chemicals, materials, and laboratory apparatus, and particle analysis using widely available flow cytometry equipment was sufficient for assay development and screening. Radical-mediated polymerization is mild and amenable to incorporating a variety of acryloyl and methacryloyl moieties. We demonstrated some of the most common functionalities deployed in chemical library synthesis, but the scope of available materials and applications is vast. For example, methacrylated gelatins and collagens promote cellular adhesion,^40,41^ PEG diacrylate-derived polymers resemble supports for synthesis of hydrophobic peptides, and polymers of *N*-isopropylacrylamide exhibit interesting thermally induced phase transitions.^42^

The magnetic hydrogel particles also alleviate solid support handling issues, introducing important advantages for automating encoded library synthesis and screening. Split-and-pool tactics underpin the current on-DNA DEL synthesis workflow, which is readily automated using routine liquid handling of interleaved aqueous building block coupling and ethanol precipitation steps. Solid-phase split-and-pool synthesis, however, is notoriously fraught; the particles aggregate and suspension handling is difficult due to settling and clogging. Magnetic particle manipulation, like robotic liquid handling, is established automation science and is routinely employed in genomic analysis workflows. Additionally, hydrogel particle deformability and freedom from aggregation could also be highly advantageous for microfluidic automation, such as droplet-scale sample preparation and screening.^14,17,22,23^

While prototyping the more complex mRNA display encoded library paradigm, we discovered that gel particles uniquely enable proximity-driven biochemical synthesis. Products of transcription from bead-bound DNA templates and subsequent translation of these gel-bound RNA transcripts are synthesized and remain concentrated only in the origin particle. High effective concentration of both DNA and RNA species in concert with the greatly reduced diffusion coefficient afforded by in-gel RNA hybridization and puromycin trapping were sufficient; no additional compartmentalization was necessary.

The proximity-driven reformatting of selection-type mRNA display libraries to bead libraries introduces well-known advantages and disadvantages of the OBOC approach. The resulting monoclonal particles are analogous to OBOC-type library beads, unlocking the possibility of functional assays. Functional screening could be advantageous for mRNA display and other enzymatically synthesized or templated libraries. Like DEL, affinity selection is the exclusive mode of ligand discovery; target binding is assumed to be a proxy for biological activity, which can only be determined after synthesis and evaluation in a formal activity assay. Unlike DEL hits, synthesis of such library members (cyclic peptides, oligonucleotides) can be intensive; thus, upfront functional screening would be highly useful for hit triaging alone. The possibility of directly evaluating more complex cellular activity is also highly desirable and uniquely the purview of the OBOC approach.^12^ Also, like OBOC technology, it is not possible to purify the bead-bound synthesis products for screening. Thus, it may be advantageous to contemplate quality control (QC) bead strategies, wherein larger and higher-capacity QC beads are doped into the library synthesis, then harvested by filtration and individually analyzed to match DNA sequences with corresponding translated peptide mass spectra, analogous to our solid-phase OBOC DEL QC strategy.^13^

OBOC library screening typically involves high-capacity synthesis resin to achieve requisite local concentrations of one library member for assay. Chemical synthesis methods applied to the gel particles resulted in high loads over a wide range (0.02–20 mM). Even incomplete conversion of RNA templates (50 uM) yielded translated peptide product concentrations of ∼100 nM. For chemotypes like conventional DEL members, which tend to be Rule-of-5 compliant,^5,6^ hit finding requires higher concentrations. However, cyclic peptides and oligonucleotides tend to emerge directly from selection with ∼1–10 nM affinities;^4^ a screening concentration of 100 nM would be sufficient to identify active species, particularly if the assay were conducted in the bead itself.^31^ Moreover, the templated OBOC synthesis approach disclosed here makes selection-to-screen workflows experimentally tractable as selection output can be used directly as input to emPCR for gel library production.

In conclusion, we describe a straightforward and scalable approach to generating hydrogel particles for encoded library synthesis. These library particles do not require additional compartmentalization in plates or droplets and are directly screened using commercial flow cytometry instrumentation. Preparation scale (10^8^–10^9^ particles) and analysis scale (10^6^ particles) suitably bridge the divide between affinity selection library sizes and conventional microplate-based functional screening, which would ordinarily require costly and low-throughput library member synthesis at scale. Although the numerical diversity is lower, the OBOC format could unlock functional screening capabilities and simultaneously enable SAR profiling of smaller scaffolds that would otherwise be difficult or impossible to isolate in affinity maturation. Key next experiments will deploy activity-based probes^43^ to detect biochemical or cellular activity perturbation with an enzymatically synthesized ligand in the hydrogel environment, which is distinct from either surface presentation or dilute solution of ligand and perhaps closer to the macromolecular crowding of the cytoplasm. Such screening capabilities could greatly expand and accelerate hit-to-lead optimization for encoded libraries.

## Supporting Information Available

Experimental details, particle sizing data, high-throughput DNA sequencing data summary, modified headpiece DNA characterization, HiBiT quantitation standard curve, AzK FACS scatterplot, and single-bead qPCR of library preparation (PDF)
