# Supplementary material for: Hydrogel-Encapsulated Beads Enable Proximity-Driven Encoded Library Synthesis and Screening
Source: ACS Cent Sci. 2023 Jul 13;9(8):1603–10. doi: 10.1021/acscentsci.3c00316 (PMC10451030; doi:10.1021/acscentsci.3c00316)
Supplement: Supplementary file 1 — oc3c00316_si_001.pdf [file oc3c00316_si_001.pdf]

Supporting Information: Hydrogel-Encapsulated Beads Enable Proximity-Driven Encoded Library Synthesis and Screening

Valerie Cavett,[†] Alix I Chan,[‡] Christian N. Cunningham,[*,‡] and Brian M. Paegel[*,¶,§]

†*Department of Pharmaceutical Sciences, University of California, Irvine*

‡*Department of Peptide Therapeutics, Genentech, South San Francisco, California 94080*

¶*Department of Pharmaceutical Sciences, University of California, Irvine, California 92697, United States*

§*Departments of Chemistry & Biomedical Engineering, University of California, Irvine, California 92697, United States*

E-mail: cunningham.christian@gene.com; bpaegel@uci.edu

Materials

All reagents were purchased from SigmaAldrich (St. Louis, MO) except where noted. N-(3-aminopropyl)methacrylamide (APMA), methacrylic acid, bovine serum albumin (BSA), light mineral oil (BioReagent), propargylamine, propargyl methacrylate, ascorbic acid, T7 RNA polymerase (NEB, Ipswitch, MA), *Taq* DNA polymerase (*Taq*, NEB), dNTPs (NEB), NTPs (NEB), Thermostable Inorganic Pyrophosphatase (TIPP, NEB), T4 DNA lig-

ase (NEB), DNase I (NEB), PURExpress in vitro transcription translation system lacking release factors (PURExpress ΔRF, NEB), acrylamide monomer solution (40% 19:1, mono:bis, Bio-Rad, Hercules, CA), ammonium persulfate (Bio-Rad), TEMED (Bio-Rad), DMF-A-6cs (Shin-Etsu of America, Akron, OH), KF-6038 (Shin-Etsu), KF-6012 (Shin-Etsu), 5-azidopentanoic acid (Chem-Impex, Wood Dale, IL), tris-hydroxypropyltriazolylmethylamine (THPTA, Chem-Impex), 3-(4-((bis((1-(tert-butyl)-1H-1,2,3-triazol-4-yl)methyl)amino)methyl)-1H-1,2,3-triazol-1-yl)propan-1-ol (BTTP, Click Chemistry Tools) Dynabeads (M-270 COOH, ThermoFisher Scientific, Waltham, MA), anti-FLAG antibody (mAb-143-AF647, ThermoFisher), CFR488 anti-V5 Tag Polyclonal Rabbit Antibody (20440, Biotium, Fremont, CA), APC anti-HA.11 Epitope Tag Antibody (901523, BioLegend, San Diego, CA), Alexa Fluor 647 N-hydroxysuccinimidyl ester (NHS-AF647, ThermoFisher), Alexa Fluor 647 alkyne (alkyne-647AF, ThermoFisher), HiBiT assay reagents (N401C and N113A, Promega), HiBiT synthetic peptide (Discovery Peptides, crb1001507j), acrylic acid N-hydroxysuccinimidyl ester (Acros Organics), argon (99.999% UHP, Praxair), and non-fat dry milk (Lab Scientific bioKEMIX, M0841) were used as provided.

Buffers and oils

Bead buffer (10 mM Tris pH 8.3, 100 mM NaCl, 0.5 mM EDTA, 0.05% KF-6012), breaking buffer (10 mM Tris pH 7.5, 100 mM NaCl, 1 mM EDTA, 1% SDS, 1% Triton X-100), lysis buffer (50 mM MOPS, 500 mM NaCl, 0.5% Triton X-100, 25 mM imidazole, 0.1 mg/mL lysozyme) and saline sodium citrate buffer (2X SSC, 30 mM sodium citrate pH 7.0, 0.5% w/v SDS, 0.3 M NaCl) were prepared in DI H_2O. Oil for emulsification (76% DMF-A-6cs, 20% mineral oil, 4% KF-6038, w/w/w) was prepared by combining materials and mixing with gentle nutation (1 h, RT, 14 rpm).

Oligonucleotides

Oligonucleotides (Integrated DNA Technologies, Inc., Coralville, IA) were obtained as desalted lyophilates and used without additional purification. 5′ exonuclease assay probes were HPLC purified at the manufacturer.

Primer Name	Description	Sequence (5′ - 3′)
P1	reverse primer	AGACCGAGATAGGGTTGAGTGTTG
ac-P1	acrydite P1	/5Acryd/AGACCGAGATAGGGTTGAGTGTTG
P2	forward primer	TGCGTCCGGCGTAGAGGATC
FAM-P1′	P1 probe	/56-FAM/CACTCAACCCTATCTC
P3	mRNA probe	/56-FAM/CTTGTCGTCATCGTCTTTGTAGTC
P4	puromycin linker	GTGTTGCCGCCCCCCGTC/iSp18//iSp18//iSp18//3Puro/
qPCR probe	qPCR probe	/56-FAM/TATATCTCC/ZEN/
		TTCTTAAAGTTAACCCTATAGTGAGTCG/3IABkFQ/
HDNA	headpiece DNA	/5Phos/GAGTCA/iSp9//iUniAmM//iSp9/TGACTCCC
L(+)	ligation (+)	GGGCCGCC/iFluorT/TCGTCCTTCTCAGCGAC
L(-)	ligation (-)	/5Phos/CCATGTCGCTGAGAAGGACGAAGGCGGCCCGG

Gene Sequences

FASTA files for the translated HiBiT epitopes are provided.

V5

TGCGTCCGGCGTAGAGGATCCTAGTAATACGACTCACTATAGGGTTAACTTTAA
GAAGGAGATATACATATGGGTAAGCCTATCCCTAACCCTCTCCTCGGTCTCGATT

CTACGGGAGGTGGTGGAAGTGTCAGCGGCTGGCGGCTGTTCAAGAAAATCAGTT
AGGACGGGGGGCGGCAACACGGCTCGAGCAACACTCAACCCTATCTCGGTCT

HA

TGCGTCCGGCGTAGAGGATCCTAGTAATACGACTCACTATAGGGTTAACTTTAA
GAAGGAGATATACATATGTACCCATACGATGTTCCAGATTACGCTGGAGGTGGT
GGAAGTGTCAGCGGCTGGCGGCTGTTCAAGAAAATCAGTTAGGACGGGGGGCG
GCAACACGGCTCGAGCAACACTCAACCCTATCTCGGTCT

FLAG

TGCGTCCGGCGTAGAGGATCCTAGTAATACGACTCACTATAGGGTTAACTTTAA
GAAGGAGATATACATATGGACTACAAAGACGATGACGACAAGGGAGGTGGTG
GAAGTGTCAGCGGCTGGCGGCTGTTCAAGAAAATCAGTTAGGACGGGGGGCGG
CAACACGGCTCGAGCAACACTCAACCCTATCTCGGTCT

Myc

TGCGTCCGGCGTAGAGGATCCTAGTAATACGACTCACTATAGGGTTAACTTTAA
GAAGGAGATATACATATGGAACAAAAACTCATCTCAGAAGAGGATCTGGGAGG
TGGTGGAAGTGTCAGCGGCTGGCGGCTGTTCAAGAAAATCAGTTAGGACGGGG
GGCGGCAACACGGCTCGAGCAACACTCAACCCTATCTCGGTCT

S-Tag

TGCGTCCGGCGTAGAGGATCCTAGTAATACGACTCACTATAGGGTTAACTTTAA
GAAGGAGATATACATATGAAAGAAACCGCTGCTGCTAAATTCGAACGCCAGCA
CATGGACAGCGGAGGTGGTGGAAGTGTCAGCGGCTGGCGGCTGTTCAAGAAAA

TCAGTTAGGACGGGGGGCGGCAACACGGCTCGAGCAACACTCAACCCTATCTC
GGTCT

NNK_5 Library template

TGCGTCCGGCGTAGAGGATCCTAGTAATACGACTCACTATAGGGTTAACTTTAA
GAAGGAGATATACATATGNNKNNKNNKNNKNNKGGAGGTGGTGGAAGTGTCA
GCGGCTGGCGGCTGTTCAAGAAAATCAGTTAGGACGGGGGGCGGCAACACGGC
TCGAGCAACACTCAACCCTATCTCGGTCT

NNT_5 Library template

TGCGTCCGGCGTAGAGGATCCTAGTAATACGACTCACTATAGGGTTAACTTTAA
GAAGGAGATATACATATGNNTNNTNNTNNTNNTGGAGGTGGTGGAAGTGTCA
GCGGCTGGCGGCTGTTCAAGAAAATCAGTTAGGACGGGGGGCGGCAACACGGC
TCGAGCAACACTCAACCCTATCTCGGTCT

Azidolysine (AzK) Peptide

TGCGTCCGGCGTAGAGGATCCTAGTAATACGACTCACTATAGGGTTAACTTTAA
GAAGGAGATATACATATGGGTGGCCACGGTGGCCATCAGCACGGCGGAGGTGG
TGGAAGTGTCAGCGGCTGGCGGCTGTTCAAGAAAATCAGTTAGGACGGGGGGC
GGCAACACGGCTCGAGCAACACTCAACCCTATCTCGGTCT

Methods

Azido oligonucleotide preparation. Azide modification of 5′-NH_2-oligonucleotides and subsequent coupling to propargyl-modified 2.8-μm Dynabeads was conducted as previously reported.[S1]

Methacrylamide DNA headpiece (ac-HDNA) functionalization. Methacrylic acid (100 µmol) was added to NHS (100 µmol) and EDC (100 µmol) in DMF and the esterification reaction incubated (5 min, RT). Methacrylic acid NHS ester solution (1 M, 15 µL) was added to HDNA (100 nmol) in phosphate buffer (0.4 M, pH 7.9), the reaction was incubated (1.5 h, RT) and then quenched with the addition of Tris buffer (20 µL, 1 M). The methacrylamide-HDNA (ac-HDNA) was purified by reversed-phase HPLC purification (Waters XTerra C18, 2.5 µm, 10 mm × 50 mm) with gradient elution (mobile phase A: 97.5% 50 mM TEAA pH 8; mobile phase B: ACN; 2.5% B 1 min, 2.5–12.5% B 20 min, 4 mL/min) and absorbance detection (260 nm). Fractions of interest were spotted (1 µL) on a MALDI-TOF MS target plate, dried, covered with THAP matrix solution (1 µL, 18 mg/mL THAP, 7 mg/mL ammonium citrate dibasic in 1:1 ACN:H_2O), dried, and analyzed via MALDI-TOF MS (Microflex, Bruker Daltonics, Inc., Billerica, MA, Fig. S3).

Hydrogel particle preparation. An aliquot of carboxylic acid-functionalized magnetic beads (M-270 carboxylic acid Dynabeads, 5×10^7, ThermoFisher Scientific) was added to 1.5-mL microcentrifuge tube and isolated magnetically. The supernatant was removed and the beads were resuspended in acrylamide monomer solution (300 µL, 0.5 M 19:1, mono:bis-acrylamide, 0.6% APS). Oil (900 µL, 4/20/76 w/w/w, KF-6038/mineral oil/DMF-A-6cs) was layered on the bead suspension. TEMED (1 µL) was added to the oil layer, the headspace in the tube was filled with Ar, and the sample was emulsified using a bead mill homogenizer (2500 rpm, 65 s, BeadBug, Benchmark Scientific, Sayreville, NJ), then incubated on ice (2 h, 4 °C). The polymerized gel particles were isolated magnetically, the supernatant was removed, and the gel particles were washed with breaking buffer (4 × 1 mL) until no trace of oil remained. Gel particles were resuspended in bead buffer (1 mL) for storage. Gel particles were variously functionalized by adding the following reagents to the acrylamide monomer solution: propargyl methacrylate (20 µM, "alkyne" function-

ality), N-(3-aminopropyl)methacrylamide (2 µM - 20 mM, ”amine” functionality), ac-P1 (20 µM, ”reverse primer” functionality), or ac-HDNA (20 µM, HDNA functionality).

Bead templating by PCR (bulk reactions, single templates). P1-functionalized magnetic microbeads (2.8-µm Dynabeads, 1×10^8) were suspended in PCR mix (0.4 mM dNTPs, 4 µM forward primer P2, 0.1 U/µL Taq polymerase in 1 × standard Taq buffer) containing DNA template (20 pg/µL) and thermally cycled ([95 °C 20 s, 60 °C 20 s, 68 °C 20 s] × 25 cycles, C1000 Touch, Bio-Rad). Beads were washed with bead buffer (4 × 1 mL) and suspended in bead buffer (1 mL) for analysis. qPCR mix (0.2 mM each dNTP, 0.5 µM each P1 and P2 primers, 0.25 µM qPCR probe, 0.05 U/µL Taq polymerase, 1 × standard Taq buffer) was prepared and aliquoted (20 µL each) to a 96-well PCR plate. Dilutions (1/100 and 1/1000) of templated bead samples were prepared in bead buffer. qPCR wells were assembled by adding diluted suspensions (1 µL). The reactions were thermally cycled ([95°C, 20 s; 60°C, 20 s; 68°C, 20 s] × 40 cycles), monitoring fluorescence (530 nm, QuantStudio3, Thermo Scientific). A standard curve was prepared using serial dilutions of the template, adding a constant volume (1 µL) to each standard reaction (100 pg/µL — 0.1 fg/µL in logs). Beads were counted by hemocytometer to obtain the average per bead template load.

Bead templating by emPCR (NNK_5 or NNT_5 library templates) P1-functionalized magnetic microbeads (1×10^8) were suspended in PCR mix (0.2 mM each dNTP, 8 µM forward primer P2, 0.02% w/v KF-6102, 0.3 U/µL Taq DNA polymerase in 1 × standard Taq buffer) containing NNK_5 or NNT_5 DNA template (1.2 fg/µL). Oil (900 µL, 4/20/76, KF-6038/mineral oil/ DMF-A-6CS, w/w/w) was added to the top of each aqueous reaction mix. The reaction was emulsified (65 s, 2500 rpm) using a bead mill homogenizer (BeadBug, Benchmark Scientific). Using a wide bore pipet tip, aliquots (50 µL) were transferred to a 96-well PCR plate and the samples were thermally cycled ([95°C, 20 s; 60°C,

20 s; 68°C, 30 s] × 35 cycles, 68°C 5 min). The plate was placed on a magnet stand and incubated (30 min), the supernatant was removed, the isolated beads were transferred in breaking buffer to a clean 1.5-mL tube, and washed with breaking buffer (4 × 1 mL). Washed beads were isolated and suspended in bead buffer (1 mL) for analysis. qPCR mix (0.2 mM each dNTP, 0.5 µM each P1 and P2 primers, 0.25 µM qPCR probe, 0.05 U/µL Taq polymerase, 1 × standard Taq buffer) was prepared and aliquoted (20 µL each) to a 96-well PCR plate. Beads were counted by hemocytometer to prepare suspensions in bead buffer of known densities (100 beads/µL and 1 bead/µL). Three 100-bead and 77 single-bead qPCR wells were assembled by adding appropriate suspension (1 µL). The reactions were thermally cycled ([95°C, 20 s; 60°C, 20 s; 68°C, 20 s] × 40 cycles), monitoring fluorescence (530 nm, QuantStudio3). A standard curve was prepared using serial dilutions of the template, adding a constant volume (1 µL) to each standard reaction (100 pg/µL - 0.1 fg/µL in logs).

In-gel oligonucleotide hybridization. Gel particles were combined with FAM-P1′ (1 µM, 20 pmol per 1 × 10^6 particles in 2 × SSC, 0.5% SDS) and incubated (2 min, RT). Gel particles were isolated magnetically, washed (2 × 200 µL, 2 × SSC, 0.5% SDS), and suspended in bead buffer (250 µL) for storage and analysis.

In-gel amine acylation. Gel particles (1 × 10^7) were resuspended (0.2 M phosphate pH 7.5, 100 µL), combined with NHS-AF647 (1 nmol), and incubated (1 h, RT). Gel particles were magnetically isolated, washed with bead buffer (2 × 200 µL), and resuspended in bead buffer (200 µL) for storage and analysis.

In-gel CuAAC. Gel particles (1 × 10^7) were resuspended in reaction buffer (1 M TEAA pH 7, 0.5% Tween-20, 100 µL) and combined with N_3-AF647 (5 nmol). Catalyst mix (50 nmol $CuSO_4$, 250 nmol ascorbic acid, 60 nmol THPTA) was added and the reac-

tion was incubated (1 h, RT). Gel particles were magnetically isolated, washed with bead buffer (2 × 200 µL), and resuspended in bead buffer (200 µL) for storage and analysis.

In-gel DNA ligation. Gel particles (2×10^6) copolymerized with ac-HDNA (20 µM) were resuspended in T4 ligase buffer (20 µL, NEB) with enzymatic ligation substrate DNA oligonucleotides (L(+) and L(-), 50 µM each), heated (2 min, 95 °C) and cooled to RT. The thermally processed reaction mixture was split in half, T4 ligase (20 U, NEB) was added to one aliquot, and the samples were incubated (1 h, RT). Gel particles were magnetically isolated, washed with bead buffer (2 × 200 µL), and resuspended in bead buffer (200 µL) for storage.

In-gel transcription. Untemplated beads labeled with 647AF and beads templated with DNA encoding the FLAG epitope (FLAG-templated beads) were encapsulated in hydrogels copolymerized with P1 (50 µM) in separate emulsion polymerizations. Cured gel particles (1×10^7 each untemplated and FLAG-templated) were suspended in T7 RNAP reaction mix (0.5 mM NTPs, 5 mM DTT, 5 U/µL T7 RNAP, 0.4 U/µL TIPP in 1 × NEB T7 buffer, 1 µM P3) and incubated (1 h, 37°C). Particles were washed with breaking buffer (2 × 500 µL) and suspended in bead buffer (400 µL) for storage and analysis.

In-gel translation (biogenic amino acids). DNA-templated beads were encapsulated in hydrogels copolymerized with ac-P1 and acrylamide modified BSA (50 µM each). BSA (2 µmol) N-acryloxysuccinimide (20 µmol) were combined in buffer (20 mM phosphate pH 7.5, 20 µL) and incubated (1 h, RT). Hydrogel particles (1×10^5 particles/µL, 10–70 µL final volume) were suspended in NEB PURExpress ΔRF reaction mix (RF1 omitted) containing puromycin capture oligonucleotide P4 (10 µM). Reactions were incubated (3 h, 37°C), washed with breaking buffer (0.5 mL), washed with PBST (0.5 mL), and suspended in PBST (10^4 particles/µL).

In-gel translation of AzK. Reagents and protocols for the in vitro translation of AzK-containing peptides were adapted from previously described methods for noncanonical amino acid incorporation.[S2,S3]

Synthesis of aminoacyl-azidolysine-CUG-tRNA^{Asn}. Aminoacylation reactions (20 µL, 20 µM tRNA and 20 µM dFx flexizyme in 0.1 M Bicine, pH 9.0) were heated (95°C, 3 min) and cooled to room temperature over 5 min. 20 mM $MgCl_2$ was added and the mixture was chilled on ice (5 min). The reaction was initiated by addition of azidolysine 3,5-dinitrobenzyl ester (25 mM in DMSO) and incubated on ice for 2 hr. After the acylation reaction, the aminoacyl-N-ε-azidolysine-CUG-tRNA^{Asn} was precipitated (0.3 M NaOAc, pH 5.2, 100% EtOH, 10,000g, 15 m). The pellet was rinsed (0.1 M NaOAc, pH 5.2, 70% EtOH) and dried.

In vitro translation of AzK-containing peptides. N-ε-azidolysine-containing peptides were translated in an genetically reprogrammed in vitro translation system from recombinant E. coli,[S2] with glutamine and GlnRS omitted to reprogram N-ε-azidolysine to the GAC codon. Briefly, in vitro translation reactions contained 50 mM HEPES pH 7.6, $Mg(OAc)_2$, 100 mM KOAc, 1 mM DTT, 2 mM spermidine, 20 mM creatine phosphate, 2 mM ATP, 2 mM GTP, 1 mM CTP, 1 mM UTP, 0.2 mM each amino acid, and 1.5 mg/mL E. coli total tRNA with final protein concentrations of 0.03 µM ArgRS, 0.09 µM GlyRS, 0.02 µM HisRS, 0.4 µM IleRS, 0.02 µM LeuRS, 0.11 µM LysRS , 0.68 µM PheRS, 0.04 µM SerRS, and 0.02 µM ValRS, 0.6 µM MTF, 2.7 µM IF1, 0.4 µM IF2, 1.5 µM IF3, 0.26 µM EF-G, 10 µM EF-Tu/Ts, 5 µM EF-P, 0.25 µM RF2, 0.17 µM RF3, 0.5 µM RRF, 1 µM T7 RNA polymerase, 3 µg/mL MK, 4 µg/mL creatine kinase, and 1.2 µM ribosome. Hydrogel particles (10^5 particles/µL, 10–30 µL final volume) were suspended in the in vitro translation mix containing puromycin capture oligonucleotide P4 (10 µM) and N-ε-azidolysine-CUG-tRNA^{Asn}.

Reactions were incubated (3 h, 37°C), washed with breaking buffer (0.5 mL), washed with PBST (0.5 mL), and suspended in PBST (10^4 particles/µL) for analysis.

In-gel CuAAC-based labeling of AzK-presenting beads. Gel particles (3×10^6) were suspended in reaction buffer (1 M TEAA pH 7, 1% BSA, 0.5% Tween-20, 100 µL) and combined with alkyne-647AF (5 nmol). Catalyst mix (25 nmol $CuSO_4$, 250 nmol ascorbic acid, 30 nmol BTTP) was added and the reaction was incubated (1 h, RT). Gel particles were magnetically isolated, washed with bead buffer (2×100 µL), and suspended in bead buffer (100 µL) for storage and analysis.

In-gel immunofluorescence detection. IVT translated particles were resuspended in detection antibody solution (4×10^4 particles/µL, 10 ng/µL APC anti-HA, 50 ng/µL CF488 anti-V5, 10% non-fat milk in PBST) and incubated (16 h, 4 °C), washed with PBST (0.5 mL) and suspended in PBST (1 mL) for flow cytometry.

Flow cytometry (particle analysis). Gel particles were analyzed by flow cytometry (NovoCyte, Agilent). Gel particles were suspected in bead buffer ($\sim 10^4$ beads/µL) for analysis. Particles were gated based on forward scatter (FSC) and side scatter (SSC) to isolate single particle populations (Fig. S2). Population fluorescence was reported for various channels (FAM $\lambda_{ex}/\lambda_{em}$ = 488/530 nm; TMR $\lambda_{ex}/\lambda_{em}$ = 561/580 nm; APC $\lambda_{ex}/\lambda_{em}$ = 640/660 nm) based on the dye label used for gel particle probing.

Confocal fluorescence imaging (particle analysis). Gel particles that were hybridized with FAM-P1′ were imaged via confocal fluorescence microscopy (Stellaris 8, Leica). Particles ($\sim 10^6$) in buffer (10 mM Tris, 100 mM NaCl, 0.05% KF-6012) were loaded into an imaging well, and allowed to settle (15 min). Particle fluorescence ($\lambda_{ex}/\lambda_{em}$ = 488/520 nm) was acquired and used to calculate object diameters (LAS).

Flow cytometry (library screening). Gel particles (5×10^6) were screened by FACS (FACSAria III, BD Biosciences). Single particle populations (gated by FSC/SSC correlation as described above) were analyzed for multi-spectral fluorescence in various channels (FAM $\lambda_{ex}/\lambda_{em}$ = 488/530 nm; APC $\lambda_{ex}/\lambda_{em}$ = 640/660 nm) based on the dye label used for gel particle probing.

HiBiT assay. Sample (5 µL solution or 2.5×10^4 hydrogels in 5 µL PBS) was combined with LgBiT/substrate mixture (5 µL, 2 × LgBiT/substrate in PBS) in a microtiter well plate (black, 384-well), and incubated (15 min, 37°C). HiBiT peptide standards (0.1–1000 nM HiBiT peptide) were similarly assembled and incubated. Luminescence was analyzed via plate reader (CLARIOstar Plus, BMG LABTECH) and unknown concentrations determined based on HiBiT standard analysis (Fig. S4).

NGS library preparation and analysis. Bead aliquots (∼1000 beads) from each of the sorted populations were subjected to PCR amplification using primers P1 and P2 and gel purified to isolate the amplicons within the range of the templates (180-220 bp for all samples). Illumina sequencing libraries were constructed using the Bioo Scientific NEXTflex Rapid DNA-Seq kit and NEXTflex unique dual index DNA barcodes (Bioo Scientific Corporation, Austin TX). Amplicon (5 ng) was end-repaired, adenylated, and ligated to adapter. The adapter-ligated product was purified via solid-phase reversible immobilization (SPRI, Agencourt AMPure XP, Beckman Coulter, Inc. Brea, CA). Adapter-ligated and purified DNA was PCR-amplified (10 cycles) and purified by SPRI (AMPure XP beads) and quantified by Kapa qPCR (Kapa Biosystems, Inc. Wilmington, MA). The libraries were denatured, diluted (12 pM final concentration), and clustered (MiSeq, Illumina, Inc., San Diego, CA) using v2 Micro SR 300 cycles chemistry and dual indexing. Analysis was performed by aligning each sequence to the control epitope or library se-

quences and counting the proportion of matches (maximum of 3 mismatches) to each aligned sequence. Raw sequence data and processing scripts are located at (TBD).

Supplemental Tables

Table S1. Hydrogels prepared by bulk emulsification scale with the size of the templating magnetic bead. Particle dispersity is inversely proportional to the magnetic bead diameter.

Bead diameter (µm)	Mean	SD	CV (%)	Gel Volume (fL)	Particle Count
1.0	4.9	1.8	36	88	447
2.8	7.5	1.9	25	260	390
10.0	12	2.0	16	570	66

Table S2. Translation and detection of non-canonical amino acid-containing gel particle library beads. NNT library particles (3×10^6) were subjected to an engineered IVT reaction that was recoded to install an azido-lysine (AzK) at CUG codons. Translated particles were washed and treated with an AF647-alkyne in a CuAAC reaction before analysis by flow cytometry. Particles were sorted to isolate the top 2% of the AF647 population and the sequences compared to the starting library. A 6-fold enrichment was observed after one round of screening.

Sample	Percentage of AzK sequences
NNT input	0.09%
sorted population (2%)	0.58%

Supplemental Figures

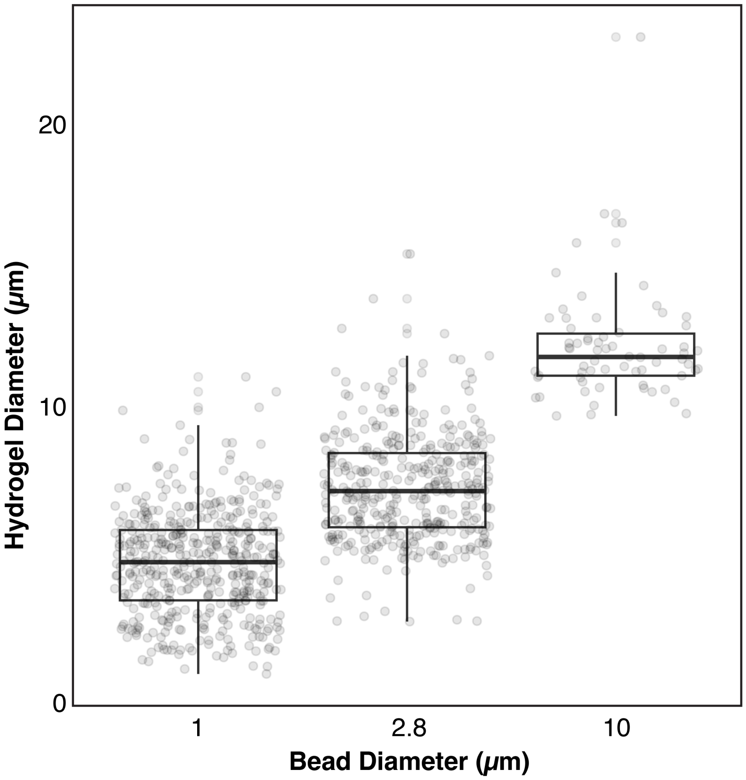

Figure S1. Hydrogels prepared by bulk emulsification scale with the diameter of the encapsualated magnetic bead. Boxes indicate the first and third quartiles; whiskers indicate the minimum and maximum observed values.

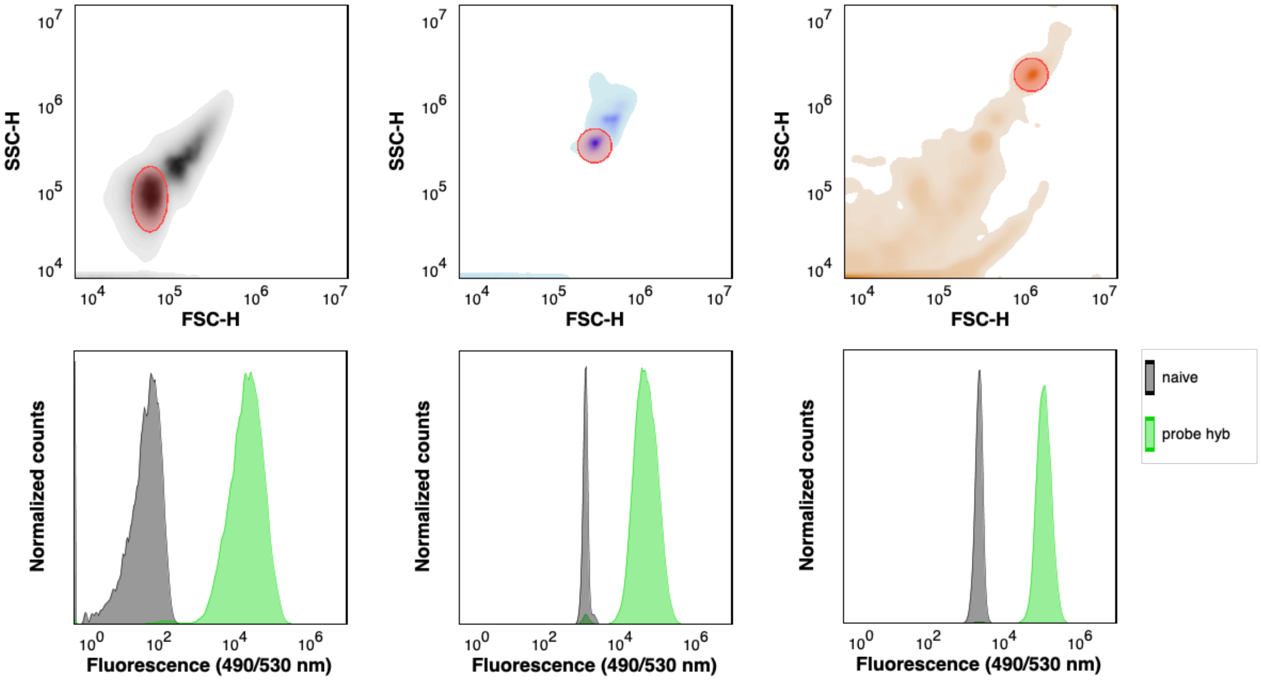

Figure S2. Hydrogels prepared around 1 (left), 2.8 (middle) or 10 µm (right) magnetic beads are analyzed by flow cytometry (top). Particle populations are gated using the forward scatter height (FSC) and side scatter height (SSC-H) parameters. Particles (grey) are baseline resolved from particles hybridized to a FAM-labeled oligonucleotide complement (green).

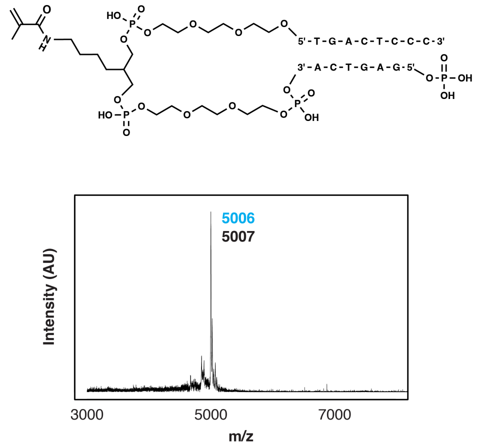

Figure S3. The primary amine of the headpiece DNA (HDNA) was acylated with methacrylic acid to yield a methacrylamide-modified oligonucleotide for copolymerization into hydrogels. The MALDI-TOF mass spectrum displays the theoretical exact mass (cyan) and observed mass (black) for the $[M+H]^+$.

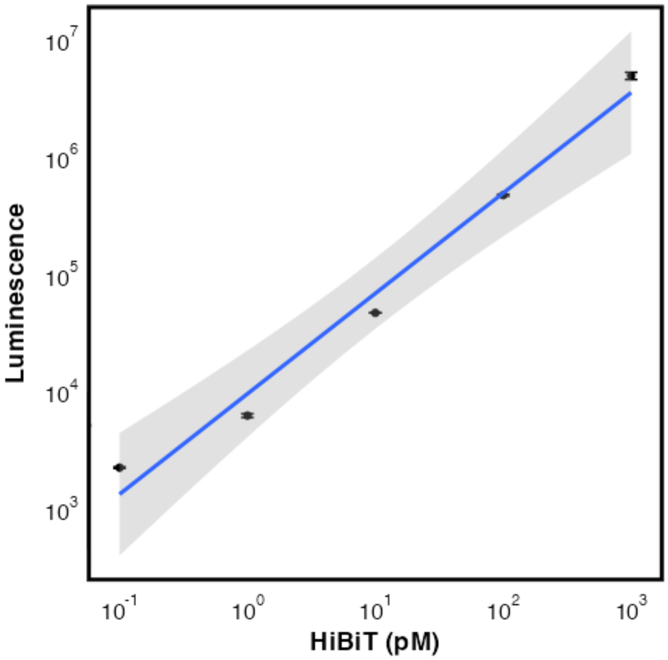

Figure S4. HiBiT peptide standards (0.1–1000 nM HiBiT peptide) were combined with LgBiT/substrate mixture (5 µL, 2 × LgBiT/substrate in PBS) in a microtiter well plate (black, 384-well), and incubated (15 min, 37 ℃). Luminescence was analyzed via plate reader and used to construct a standard curve. Unknown samples were quantitated by comparison to the standard curve.

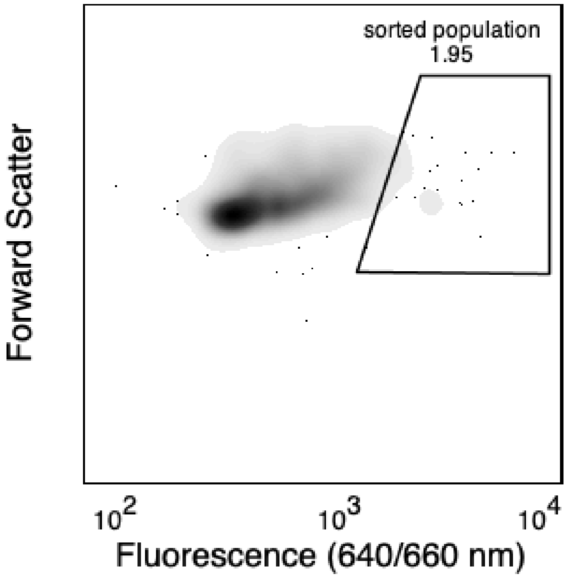

Figure S5. NNT library particles (3×10^6) prepared by emPCR were subjected to an engineered IVT reaction that was recoded to install an azido-lysine (AzK) at CUG codons. Translated particles were washed and incubated with an AF647-alkyne in a CuAAC reaction before analysis by flow cytometry. Particles were sorted by gating the top 2% of the AF647 signal.

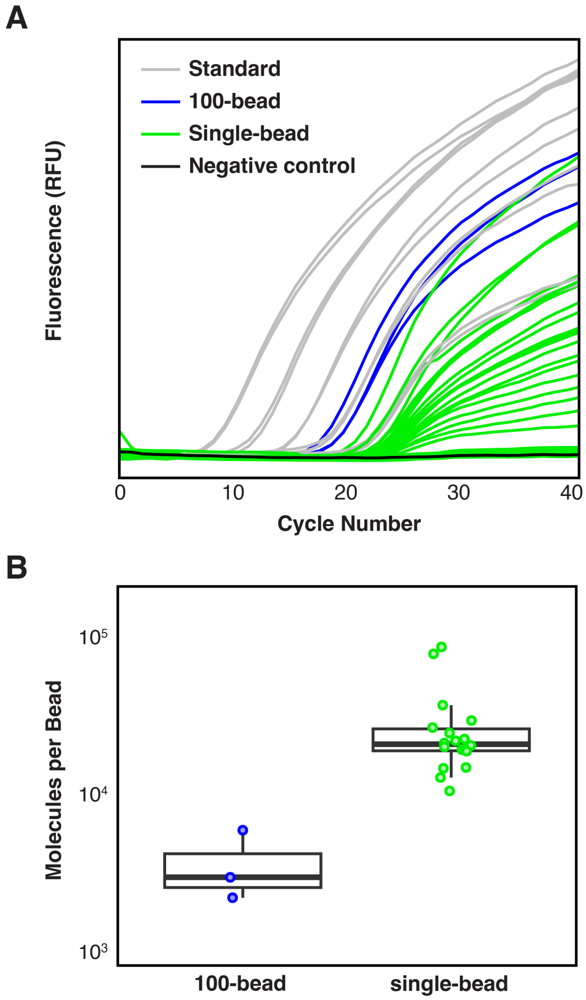

Figure S6. qPCR analysis of emPCR library preparation. (A) Aliquots of 100 beads are quantitated for to obtain average loading (blue traces) and limiting dilution of beads is sampled in 77 wells (green traces) to obtain single-bead quantitation of DNA templating. Traces for standards (gray) and negative template control (black) are shown. (B) Quantitation of 100-bead and DNA-templated single beads is shown in the box plots. Average particle loading for the 100-bead aliquot was 4,200 DNA molecules per bead. Average particle loading for single DNA-templated beads was 42,000 DNA molecules per bead.

References

(S1) Malone, M. L.; Cavett, V. J.; Paegel, B. M. Chemoselective Coupling Preserves the Substrate Integrity of Surface-Immobilized Oligonucleotides for Emulsion PCR-Based Gene Library Construction. *ACS Combinatorial Science* **2017**, *19*, 9–14.

(S2) Murakami, H.; Ohta, A.; Ashigai, H.; Suga, H. A highly flexible tRNA acylation method for non-natural polypeptide synthesis. *Nature Methods* **2006**, *3*, 357–359.

(S3) Adaligil, E.; Song, A.; Cunningham, C. N.; Fairbrother, W. J. Ribosomal Synthesis of Macrocyclic Peptides with Linear γ^4 and β^4-Hydroxy-γ^4-amino Acids. *ACS Chem. Biol.* **2021**, *16*, 1325–1331, PMID: 34270222.
